# Fuzzy logic nursing tool for early acute kidney injury detection in surgical patients

**DOI:** 10.3389/fneph.2025.1624880

**Published:** 2025-08-27

**Authors:** Nooreena Yusop, Samsiah Mat, Ruslinda Mustafar, Muhammad Ishamuddin Ismail

**Affiliations:** ^1^ Faculty of Nursing, University College of MAIWP International (UCMI), Kuala Lumpur, Malaysia; ^2^ Department of Medicine, Faculty of Medicine, University Kebangsaan Malaysia, Bangi, Selangor, Malaysia; ^3^ Head of Cardiothoracic, Head of Heart and Lungs Centre, Hospital Canselor Tuanku Muhriz, Cheras, Kuala Lumpur, Malaysia

**Keywords:** fuzzy logic model (FLM), nursing risk assessment, acute kidney injury, predictive tool, surgical patients

## Abstract

**Background:**

Acute Kidney Injury (AKI) is a common yet preventable complication among surgical patients, contributing to increased morbidity, prolonged hospital stays, and higher healthcare costs. Early detection is critical; however, the absence of a standardized nursing-led risk assessment tool for AKI limits proactive intervention in clinical practice.

**Objective:**

This study aimed to develop and evaluate the Nursing Risk Assessment for Acute Kidney Injury tool, integrating the Fuzzy Logic Model (FLM) to enhance interpretive accuracy and improve nursing-led AKI risk detection and decision-making.

**Methods:**

A Design and Development Research (DDR) framework was employed in three phases. Phase 1 involved a needs analysis using a focus group discussion to explore the necessity of AKI assessment among surgical nurses. Phase 2 focused on tool development through expert consensus (surgeon, nephrologist, nursing academician, and experienced nurse) and evidence synthesis via a systematic literature review. In Phase 3, the Nursing Risk Assessment-AKI tool was evaluated through a quasi-experimental design at Hospital Canselor Tuanku Muhriz (HCTM), Kuala Lumpur, involving 75 surgical nurses assessing 200 patients.

**Results:**

Post-intervention analysis indicated increased nursing confidence, with 95.7% expressing positive perception of tool use. The FLM-supported tool demonstrated a predictive accuracy of 81.3%; however, the potential for false positives or negatives remains, especially given the single-center context. Fuzzy logic stratified patients into risk groups: at risk (33.5%), borderline (20.5%), and no risk (46.0%). ANOVA analysis revealed significant differences (p < 0.05) between AKI risk and factors such as age, gender, comorbidities, clinical/laboratory parameters, surgery types, and nephrotoxic agent usage.

**Conclusion:**

While initial findings support the usability and clinical feasibility of the NURA-AKI tool, further multicenter validation is needed. The tool is designed to complement nurse judgment, promoting early AKI detection and structured risk communication in surgical care without replacing clinical autonomy.

## 1 Introduction

Acute kidney injury (AKI) is a common complication among surgical patients, contributing to higher postoperative mortality and prolonged hospital stays (1)​. Because no specific pharmacotherapy exists to reverse established AKI, early risk assessment and preventive interventions are paramount in improving patient outcomes​ (2). Innovative nursing tools offer a promising avenue to strengthen early detection of AKI. For example, point-of-care decision aids like mobile applications have significantly improved timely AKI recognition and enabled earlier interventions, translating into better patient recovery​ (3). Harnessing such technologies – including advanced models like fuzzy logic systems – could empower nurses to anticipate AKI risk more effectively and initiate prompt protective measures.

Despite the clinical importance of early AKI detection, there is currently a lack of nursing-specific methods or protocols for proactive risk assessment. Nurses often rely on standard postoperative monitoring and physician alerts that identify AKI only after kidney function has notably declined​ (4). This reactive approach means that opportunities for early intervention are frequently missed. For instance, the gold-standard diagnostic marker (serum creatinine) can take 24–36 hours to rise after renal injury​ (5) by which time damage may have progressed. The absence of a streamlined, nurse-driven risk assessment tool for AKI represents a critical gap in perioperative care.

Existing AKI risk stratification models and scores have notable shortcomings when applied in diverse clinical settings. Many traditional prediction tools (e.g., post-cardiac surgery risk scores) were developed using linear regression techniques and a limited set of variables, which do not adequately capture the complex, nonlinear interactions of patient risk factors​ (6, 7). Consequently, these models have often failed to meet expectations in real-world use, showing suboptimal predictive performance and low adoption in practice​ (8, 9). The reliance on static risk equations and delayed indicators hampers clinicians’ ability to dynamically identify high-risk patients. This limitation underscores the need for more robust and flexible risk assessment approaches that can be better integrated into routine nursing assessments.

Given the above gaps, there is a growing imperative to integrate intelligent decision-support systems into clinical practice for AKI prevention. Advanced technologies like artificial intelligence (AI) have demonstrated the ability to monitor patients continuously and flag subtle physiologic changes earlier than human observation alone, enabling timelier interventions (10)​. Fuzzy logic–based models in particular offer an approach to handle the ambiguity and “fuzzy” boundaries often present in clinical data, which conventional scoring systems struggle with (11).​ Incorporating such intelligent tools into nursing workflows could provide real-time risk alerts and guidance, supporting nurses’ clinical judgment with data-driven insights. This addresses current shortfalls by personalizing risk stratification and ensuring that at-risk surgical patients are identified and managed before AKI progresses.

This study contributes to the advancement of nursing practice by developing a novel tool that enhances nurses’ capacity for early AKI risk identification. Integrating a fuzzy logic model into nursing assessments, allowing nurses to detect patient deterioration that might otherwise go unrecognized until AKI is established. Similar AI-driven monitoring systems have already shown that empowering nurses with early warnings leads to timely interventions that reduce complications and improve patient outcomes (10, 12). By adopting such technology, nursing practice can shift from a reactive to a proactive stance in AKI prevention, reinforcing the nurse’s role in safeguarding surgical patients’ renal health (13, 14).

The proposed research also strengthens evidence-based practice in nursing risk assessment. The fuzzy logic risk tool will be built upon validated risk factors and clinical data, ensuring that its alerts and stratifications are grounded in current evidence rather than anecdotal experience. Point-of-care tools like this facilitate consistent application of evidence-based criteria; for example, prior studies have shown that decision-support apps improve adherence to AKI diagnostic guidelines and enable earlier detection aligned with best practices (15, 16). By standardizing how risk is evaluated, the tool helps nurses make clinical decisions that are transparent and reproducible. Ultimately, this elevates the quality of care by marrying nurses’ clinical expertise with data-driven, evidence-backed risk assessments (10).

Implementing a fuzzy logic–driven AKI risk assessment aligns with broader healthcare digitalization and smart hospital initiatives. Healthcare systems worldwide are rapidly adopting AI and digital tools to enhance patient care, and nursing must evolve in tandem (17). The development of an interpretable AI tool for AKI is in step with this trend, leveraging electronic health data and machine intelligence to improve clinical decision-making at the bedside. Notably, the spread of electronic health records and machine learning techniques has already brought new potential to predictive modeling in AKI care​ (18). However, while FLMs may enhance interpretive flexibility, their successful implementation depends on factors beyond algorithmic accuracy. Barriers such as the need for proper nurse training, risks of over-reliance, false alerts, and integration with existing workflows must be critically addressed. Without adequate user education, human oversight, and institutional support, even advanced tools may fail to gain adoption or may inadvertently compromise clinical autonomy.

This study aims to develop and interpret a nursing-led AKI risk assessment tool using a fuzzy logic model. Specifically, the goal is to design a fuzzy inference system (the NURA-AKI tool) that can evaluate postoperative patients’ risk of AKI in real-time and to provide an interpretable output that nurses can readily understand and act upon. By leveraging fuzzy logic’s capacity to handle uncertain or imprecise clinical inputs​ (19, 20), the tool will mimic expert clinical reasoning in estimating AKI risk. The research also seeks to interpret the model’s decision rules and output – for example, by identifying which patient factors most strongly influence the risk calculation – to ensure the tool’s recommendations are transparent and clinically informative (21). The goal of this study is to create a decision-support mechanism that complements, rather than replaces, nursing judgment. By embedding fuzzy inference into routine nursing workflows and aligning tool outputs with evidence-based clinical parameters, the research aims to balance innovation with feasibility, supporting the broader agenda of safe and ethical AI integration in nursing practice.

## 2 Material and method

### 2.1 Development of nursing risk assessment of AKI tool

The development of the Nursing Risk Assessment for Acute Kidney Injury tool was guided by a systematic Design and Development Research (DDR) approach, integrating evidence-based frameworks to enhance AKI risk detection among surgical patients (22, 23). The process began with a comprehensive literature review, expert input from surgeons, nephrologists, and renal nursing specialists, and continuous refinement to ensure the tool’s practicality and clarity for clinical application. Drawing from the Acute Dialysis Quality Initiative (ADQI) framework, the NURA-AKI form categorizes AKI risk factors across perioperative, intraoperative, and postoperative phases, emphasizing the dynamic interplay of renal hypoperfusion and oxygen imbalance leading to AKI development (24, 25).

The risk factors embedded in the tool include demographic factors (age >65 years, male gender), comorbidities (hypertension, diabetes, CKD, liver disease, CVS), intraoperative challenges (hypotension, blood loss, prolonged surgery), risk factor surgery (emergency/major surgery/involving cardiac procedure) and consumption of nephrotoxin agent including radiocontrast agent (26). This structure allows for holistic and systematic assessment, promoting early identification and preventive interventions.

The theoretical foundation of the NURA-AKI form is further strengthened by Donabedian’s Structure-Process-Outcome (SPO) model, recognizing that healthcare quality results from the interplay of systemic structures (nurses’ knowledge and resources), clinical processes (systematic AKI risk assessment using NURA-AKI), and patient outcomes (reduction in AKI incidence). This alignment ensures the tool’s integration into standard nursing practice, emphasizing education, early detection, and outcome monitoring to improve patient safety (27).

To enhance decision-making accuracy, the Fuzzy Logic Model (FLM) was adapted into the NURA-AKI form. Unlike rigid binary assessments, FLM accommodates the complexity of clinical data through membership functions, fuzzy sets, and linguistic variables. These adaptations allowed for FLM to work with interval inputs, which are often encountered in healthcare. FLM operates with fuzzy sets, where an element’s membership in a set can be ambiguous or indefinite, a common scenario in health-related data. It leverages concepts like fuzzification, defuzzification, membership functions, linguistic variables, and domain rules to arrive at conclusions or outcomes (20). It allows for detailed interpretation of AKI risk based on clinical parameters such as serum creatinine, albumin, hemoglobin, fluid loss, and nephrotoxic medication use (21, 28).

**Figure 1:**
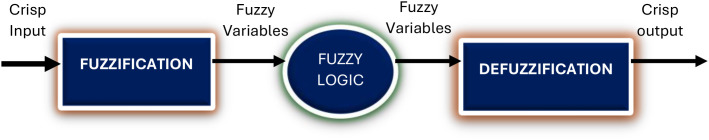
Application the concept of Fuzzy Logic Model (20).

**Figure 1**: Application the concept of Fuzzy Logic Model (20).

Fuzzy rules in a Fuzzy System play a crucial role in determining the system’s behavior and decision-making process. In the context of detecting Acute Kidney Injury (AKI) for surgical patients, these rules are designed to capture the complex and imprecise relationships between various clinical parameters and the likelihood of AKI occurrence. Fuzzy rules are typically expressed using linguistic variables and membership functions to allow for a more detailed and context-aware assessment.

**Figure 1** illustrates the Fuzzy Logic Model (FLM) framework applied to support nurse-led detection of Acute Kidney Injury (AKI) in surgical patients. The process begins with crisp input values—measurable clinical and laboratory parameters such as serum creatinine, urine output, nephrotoxin exposure, type of surgery, and existing comorbidities. These inputs are fuzzified using linguistic variables (e.g., “low,” “moderate,” “high”) that are mapped to membership functions. This allows each variable to be interpreted on a continuum, accommodating clinical uncertainty and borderline findings. In this phase, the researcher applies Fuzzy Rules to detect AKI for surgical patients. These rules were developed in consultation with clinical experts and based on evidence-informed thresholds.

The fuzzy inference system then applies a structured set of if–then rules, as detailed in **Table 1**, to assess the relationship between multiple risk factors and the likelihood of AKI. For example:

- If serum creatinine is elevated AND nephrotoxin exposure is present AND patient has sepsis, then AKI risk is high.
  - If urine output is borderline AND comorbidities are mild, then AKI risk is borderline.

**Table 1:** Application of Fuzzy rules to detect AKI for surgical patients.

Rule 1	Age of patient ≥65 years; if patient age more than 65 years old, patient is at risk of AKI
Rule 2	Male; if patient is male, patient is at risk of AKI.
Rule 3	Risk factor comorbidity; if patient has one of any following comorbidity e.g hypertension/ hypotension/ diabetes/ chronic liver disease/ cardiovascular disease/ or chronic kidney disease (e-GFR < 60ml/min per 1.73m^2^, patient is at risk of AKI
Rule 4	Risk factor Clinical and Laboratory result; if patient has one of any clinical/ laboratory parameters such as dehydration/ blood loss/ Serum Creatinine < 26.5mmol/L within 48hours/ hypoalbuminemia (< 34g/ dL) or hyperalbuminemia (> 50g/dL)/ hyponatremia (< 135mmol/L) or hypernatremia (> 145mmol/L)/ Anemia (< 10g/dL), Urine output (< 0.5ml/kg/hour for 6 hours) or Proteinuria (> 80g/dL); patient is at risk of AKI
Rule 5	Risk factor Surgery; if patient has one of any risk factor surgery e.g duration elective surgery > 3hours/ emergency surgery/ major surgery or involving cardiac procedure, patient is at risk of AKI.
Rule 6	Risk factor nephrotoxin medication; if patient has consumed any nephrotoxin including radiocontrast agent, patient is at risk of AKI

Fuzzy rules were developed to classify patients into “At Risk,” “Borderline,” or “No Risk” categories based on the number and percentage of identified risk factors. The fuzzy approach allows healthcare providers to interpret AKI risk more realistically, acknowledging that many risk factors exist on a continuum rather than as absolutes. This enables personalized patient monitoring and intervention strategies, essential for optimizing postoperative outcomes.

**Figure 2:**
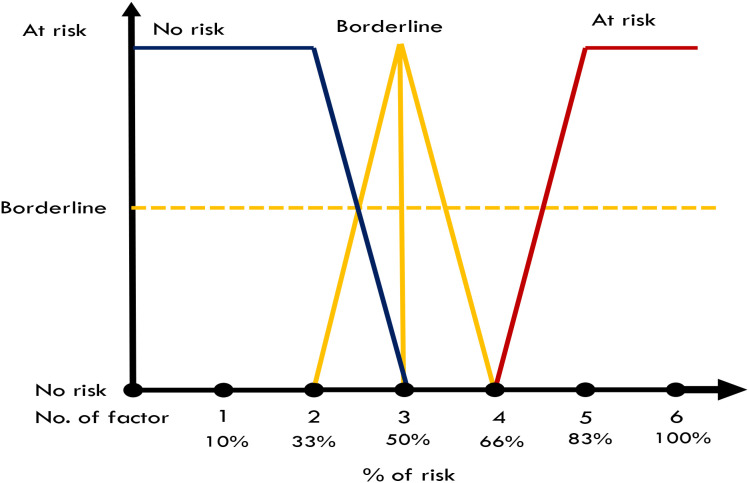
The Fuzzy Set Membership Function to interpret risk of AKI.

The final risk interpretation framework classified patients as “At Risk” if they presented with four or more risk factors (≥66% risk), “Borderline” with three factors (50% risk), and “No Risk” with one to two factors (<33% risk) as shown in **Table 2**. This stratification enhances the precision of clinical decision-making, ensuring timely preventive measures for patients most vulnerable to AKI. As shown in **Figure 2** it presents the rule base that governs the NURA-AKI system’s reasoning process. The tool aggregates the number and percentage of active AKI risk factors and assigns a corresponding fuzzy output: “No Risk,” “Borderline,” or “At Risk. This output is defuzzified into a crisp numerical value (0–1), enabling stratified risk scoring. By incorporating this fuzzy rules-based approach, the NURA-AKI tool offers a flexible, interpretable, and nurse-friendly system that mimics expert clinical judgment and enhances real-time decision-making.

**Table 2:** The Interpretation of AKI risk according to the number of risk factor.

No. of risk factor	Percentage (%) of risk	Risk of AKI interpretation
6	100%	At risk
5	83%	At risk
4	66%	At risk
3	50%	Borderline
2	33%	No risk
1	10%	No risk

Overall, the development of the NURA-AKI form supported by FLM principles marks a significant step forward in nursing-led AKI risk assessment. It provides surgical nurses with a user-friendly, evidence-based tool that improves early AKI detection, promotes proactive patient care, and strengthens healthcare outcomes in surgical settings. The Fuzzy Logic Model (FLM) integrated within the NURA-AKI tool is purposefully designed as a clinical decision-support system, not as a diagnostic authority, reinforcing the principle that nurse autonomy and clinical judgment remain paramount in-patient care. The tool provides structured, algorithm-guided insights to assist nurses in identifying patterns suggestive of acute kidney injury (AKI) risk, particularly in perioperative settings where early detection is critical (29). Recognizing the potential for over-reliance or misinterpretation, a structured training and education module was implemented to equip nurses with the knowledge and confidence to appropriately interpret NURA-AKI outputs (30). The model’s alerts particularly those categorized as “At Risk” or “Borderline” which are designed to act as clinical prompts rather than absolute directives, encouraging further patient assessment and, where necessary, physician consultation. To mitigate the risk of false positives, nurses are trained to correlate NURA-AKI outputs with other clinical and laboratory parameters, reinforcing critical thinking. This safeguard ensures that decision-making remains holistic and patient-centred (31). Furthermore, nurses’ clinical independence is emphasized throughout tool implementation, with protocols underscoring the supportive nature of the tool in enhancing but not to override the professional judgment. The deployment of NURA-AKI is also accompanied by ongoing monitoring and feedback loops, where nurse experiences and outcomes are reviewed periodically to ensure the tool is used effectively and safely. This reinforces a culture of reflective practice, adaptive learning, and ethical AI integration in nursing, aligning with both clinical governance and patient safety priorities. Ongoing monitoring and feedback foster reflective practice and ethical AI integration aligned with patient safety priorities (32, 33).

### 2.2 Validity of nursing risk assessment of AKI tool

The process of validating a research instrument, like the Nursing Risk Assessment of AKI tool, is an indispensable undertaking aimed at guaranteeing its precision in measuring the intended parameters. This validation journey typically encompasses two pivotal dimensions of assessment: face validity and content validity.

#### 2.2.1 Face validity

Face validity is the initial impression that a research instrument gives to respondents or experts who examine it. In the case of the Nursing Risk Assessment of AKI tool, face validity aims to assess whether the form appears to measure what it is designed to measure, which is the risk of Acute Kidney Injury (AKI) in surgical patients. The face validity process often involves presenting the instrument to a group of experts, such as surgeon (ID01), nephrologist (ID02), nursing academician (ID03) and experienced nurse (ID04).

The face validity assessment of the NURA-AKI form revealed that ID01 (Surgeon) provided a negative rating to the item statement “Risk factor of electrolyte imbalance” concerning the “Reasonableness of item in relation to the supposed purpose of the instrument.” In light of this feedback, ID01 recommended modifying the statement to “Risk factors of Clinical and Laboratory Parameters” to better convey the intended meaning accurately. Additionally, ID02 (Nephrologist) rated negatively to the item “CKD” in terms of the appropriateness of the statement. To address this concern, ID02 suggested refining the statement to “Chronic Kidney Disease (e-GFR < 60ml/min per 1.73m²)” for greater precision. These expert-driven refinements aim to ensure the utmost accuracy, relevance, and clarity of the NURA-AKI form, aligning it more closely with the intended purpose and content validity of the instrument.

#### 2.2.2 Content validity

The content validity assessment for the Nursing Risk Assessment of AKI tool involved a panel of four healthcare experts: a nephrology academician, a cardiothoracic surgeon, an experienced critical care nurse, and a nephrology lecturer, all holding at least a master’s degree. Experts were invited via email and asked to complete an online survey evaluating the relevance of each item. They rated each item using a four-point Likert scale (0 = not relevant to 4 = highly relevant). Relevance scores were then recoded (1 for ratings of 3 or 4; 0 for ratings of 0 or 1) to compute the Item Content Validity Index (I-CVI) and the Scale Content Validity Index (S-CVI) following standard procedures.

Among the 10 items, full agreement (I-CVI = 1.00) was achieved by three experts, while the nursing lecturer rated two items (items 3 and 7) as ‘somewhat relevant’ (I-CVI = 0.75). The average scale content validity (S-CVI/Ave) was 0.95, and the universal agreement score (S-CVI/UA) was 0.80, both exceeding the acceptable thresholds for strong content validity (34, 35). These results confirmed that the NURA-AKI form was relevant, appropriate, and well-aligned with clinical practice needs (36).

### 2.3 Respondents and sampling

A total of 105 registered nurses were employed in the surgical department of Hospital Canselor Tuanku Muhriz (HCTM), Kuala Lumpur, forming the target population for this study. However, 15 nurses were excluded as they were on extended leave (e.g., maternity, study, or unpaid leave) or planning retirement or resignation during the data collection period. Using the Raosoft sample size calculator with a 5% margin of error, 95% confidence interval, and 50% response distribution, a minimum sample size of 75 nurses was determined to be statistically adequate. Consequently, 75 participants were selected through proportional stratified random sampling. Eligible participants included registered nurses—either permanent or contract staff—with at least one year of experience in the surgical department. Nurses currently posted in surgical wards were included, while temporary personnel such as nursing students and attachment nurses were excluded from the study.

Meanwhile, as for the assessed patient, nurses select patients according to specific criteria in 10 surgical units in HCTM. The assessment covered various scenarios, including new admissions, inter/intra-facility patient transfers, and patients returning from the operating theater (OT), encompassing emergency, elective, minor, and major surgeries. Exclusions were outlined for lodging patients without surgical involvement and those undergoing ongoing kidney replacement therapy.

### 2.4 Research protocol

A comprehensive research protocol was developed to ensure strict adherence to the designated timeline among surgical nurses participating in the Nursing Risk Assessment of Acute Kidney Injury program. Each participant was assigned a unique code and identification number to enable precise tracking and documentation. Invitations were extended for a Continuous Nursing Education (CNE) session, allowing participants to either attend face-to-face training or access a recorded lecture via YouTube https://youtu.be/3eDBJCnwpb0?si=TeTwEBLx4b1BMp5H to accommodate varying schedules. An AKI learning module was systematically developed, integrating multidisciplinary case studies from general surgery, neurosurgery, urosurgery, and cardiac surgery to enhance clinical application. Practical demonstrations were incorporated in both physical and online formats, equipping nurses with the skills necessary for accurate use of the Nursing Risk Assessment-AKI tool. To facilitate efficient communication, a dedicated WhatsApp group, “AKI Research Group,” was established. Following one week of the education intervention, the tool was distributed for clinical application, with assessments conducted according to standardized criteria. Subsequently, an evaluation survey was administered after two weeks to gather participant feedback on the usability and application of the tool. This structured protocol emphasized ethical conduct, participant comprehension, and flexibility, thereby ensuring rigorous training, consistent data collection, and enhanced competency in AKI risk assessment practices.

### 2.5 Ethical approval

Ethical approval for this study was granted by the Universiti Kebangsaan Malaysia (UKM) Research Ethics Committee and the Director’s Office of Hospital Canselor Tuanku Muhriz (HCTM), under reference number JEP-2022-161. Additionally, formal permission to conduct the study was obtained through a meeting with the Head of the Training and Development Unit, Nursing Department, HCTM. The study was officially approved for implementation over a 12-month period, from April 2022 to March 2023.

## 3 Results

### 3.1 Risk of AKI

Seventy-five nurses working in the surgical department who had undergone AKI and Nursing Risk Assessment education program were required to perform risk assessments for surgical patients using Nursing Risk Assessment of AKI tool. The assessment was performed based on three time series: series one assessment was performed in July 2022, series two performed in September 2022, and series three performed in December 2022 (**Table 3**).

**Table 3:** Nurses characteristic and confidence level.

Nurse’s Characteristics		Assessment 1	Assessment 2	Assessment 3
Assessment duration		(Jul-Aug 2022)	(Sept-Oct 2022)	Dec 2022-Jan2023
Nurses participation		n=68	n=70	n=62
Nurses’ age mean (SD)		35.9(5.37)	35.2(5.41)	36.1(5.38)
Nursing position				
Head nurse		9	9	9
Staff Nurse		59	61	53
Nurse’s confidence level rate (n, %)				
Not confident at all		2(2.9%)	–	–
Not really confident		4(5.9%)	2(2.9%)	–
Slightly confident		27(39.7%)	11(15.7%)	–
Neutral		18(26.5%)	4(5.7%)	–
Confident		15(22.1%)	47(67.1%)	31(50.0%)
Highly confident		2(2.9%)	5(7.1%)	22(35.5%)
Very confident		–	1(1.4%)	7(11.3%)
Extremely confident		–	–	2(3.2%)
			

The number of participating nurses were 68, 70, and 62 individuals in Assessments 1, 2, and 3, respectively. The mean age ranges from 35.9 (SD 5.37) to 36.1 (SD 5.38). Notably, nurses’ confidence levels exhibited a dynamic progression, with a substantial increase from 22.1% in Assessment 1 to 67.1% in Assessment 2, followed by a slightly decreased but sustained level at 50.0% in Assessment 3. The majority of nurses transitioned from lower to higher confidence levels, with Assessment 3 showing increased percentages of nurses reporting ‘Highly confident’ (35.5%) and ‘Very confident’ (11.3%). Overall, these findings highlight a positive trajectory in nurses’ confidence in utilizing the Nursing Risk Assessment-AKI tool for risk assessments in the surgical department. 

A total of 200 surgical patients were assessed across three phases. The mean age remained consistent: 56.9 (SD 16.6) in Assessment 1, 54.9 (SD 16.5) in Assessment 2, and 54.9 (SD 16.4) in Assessment 3. Gender distribution was balanced, while Malay patients made up the majority, though their proportion decreased over time. Chinese patient representation increased from 20.6% to 38.7%, while Indian patients also rose from 5.9% to 16.1%. ‘Other’ races were minimal and only recorded in the first two phases (see **Table 4**).

**Table 4:** Patient characteristics during assessment performed.

	Assessment 1	Assessment 2	Assessment 3
Assessment duration	(Jul-Aug 2022)	(Sept-Oct 2022)	Dec 2022-Jan2023
Patient’s characteristic	n=68	n=70	n=62
Patient’s age mean (SD)	56.9 (16.6)	54.9 (16.5)	54.9 (16.4)
Gender (n, %)			
Male	44 (64.7%)	39 (55.7%)	33 (53.2%)
Female	24 (35.3%)	31 (41.3%)	29 (46.8%)
Races			
Malay	49 (72.1%)	41 (58.6%)	28 (45.2%)
Chinese	14 (20.6%)	22 (31.4%)	24 (38.7%)
Indian	4 (5.9%)	5 (7.1%)	10 (16.1%)
Others	1 (1.5%)	2 (2.9%)	–
Admission via			
Counter admission	29 (42.6%)	24 (34.3%)	14 (22.6%)
Emergency department	22 (32.4%)	24 (34.3%)	22 (35.6%)
Operating theater	6 (8.8%)	–	5 (8.1%)
Ward	1 (1.5%)	9 (12.9%)	1 (1.3%)
Other hospitals	1 (1.5%)	2 (2.9%%)	3 (4.8%)
Clinic	9 (13.2%)	10 (14.3%)	10 (16.1%)
Critical care unit/ ICU	–	1 (1.4%)	6 (9.7%)
Semi-critical care unit	–	–	1 (1.6%)
Type of surgical procedure			
No surgery	4 (5.3%)	20 (28.6%)	13 (21.0%)
Elective	40 (53.3%)	37 (52.9%)	45 (45.2%)
Emergency	24 (32.0%)	13 (18.6%)	28 (33.9%)

Admission pathways varied, with counter admissions decreasing across phases (42.6% to 22.6%), while Emergency Department admissions remained stable (~34–35%). Clinic admissions increased slightly, and operating theater admissions dropped in Assessment 2 before rising again. Other sources (wards, critical care units, and external hospitals) remained under 15%. 

Surgical procedures also varied. Elective surgeries remained the most common (53.3% in Assessment 1, 45.2% in Assessment 3), while emergency surgeries fluctuated (32.0% in Assessment 1, 33.9% in Assessment 3). Patients not undergoing surgery increased in Assessment 2 (28.6%) before dropping to 21.0% in Assessment 3. These findings highlight the evolving demographic, clinical, and procedural patterns among surgical patients during the evaluation period.

**Table 5** presents an overview of nurses’ AKI risk assessments among 200 surgical patients. Hypertension was the most common comorbidity (57.0%), followed by Diabetes Mellitus (43.5%) and cardiovascular disease (25.0%), while hypotension and chronic liver disease were least common (8.0%). Among clinical and laboratory factors, elevated serum creatinine >26.5 mmol/L was the highest risk indicator (33.5%), followed by dehydration/blood loss and anemia (both 24.0%), hypo/hyperalbuminemia (22.5%), and reduced urine output (15.5%). Proteinuria and hyperkalemia were identified less frequently.

**Table 5:** Patients’ AKI- risk factors assessment performed by nurses (patients (n)= 200).

Risk Factors AKI	Risk of AKI		
	YES	UNSURE	NO
	n (%)	n (%)	n (%)
Patient’s comorbidity			
Hypertension	114 (57.0%)	3 (1.5%)	83 (41.5%)
Hypotension	16 (8.0%)	22 (11.0%)	162 (81.0%)
DM	87 (43.5%)	3 (1.5%)	110 (53.0%)
Chronic liver disease	16 (8.0%)	9 (4.5%)	175 (87.5%)
Cardiovascular disease	50 (25.0%)	7 (3.5%)	143 (71.5%)
Chronic kidney disease (eGRF <60ml/min per 1.73m^2^)	27 (13.5%)	27 (13.5%)	146 (73.0%)
Malignancy/tumor	41 (20.5%)	5 (2.5%)	154 (77.0%)
Sepsis	45 (22.5%)	8 (4.0%)	147 (73.5%)
Clinicaland laboratory parameters			
Dehydration/ blood loss	48 (24.0%)	20 (10.0%)	132 (66.0%)
SrCreatinine >26.5mmol/L within 48 hours	67 (33.5%)	10 (5.0%)	123 (61.5%)
Albumin level <34 or > 50g/Dl	45 (22.5%)	10 (5.0%)	145 (72.5%)
Sodium level <135 or >145mmol/L	39 (19.5%)	6 (3.0%)	158 (79.0%)
Potassium level >5.5 mmol/L	19 (9.5%)	6 (3.0%)	175 (87.5%)
Hb level <10g/Dl	48 (24.0%)	6 (3.0%)	146 (73.0%)
Urine Output < 0.5ml/kg/hour for 6 hours	31 (15.5%)	3 (1.5%)	166 (83.0%)
Protein level >80g/Dl	11 (5.5%)	5 (2.5%)	184 (92.0%)
Surgery procedure			
Elective	62 (66.0%)	9 (9.6%)	23 (24.5%)
Emergency	63 (96.9%)	1 (1.5%)	1 (1.5%)
Not performed	0 (0%)	0 (0%)	41 (100%)
Hours of surgery performed			
<3hours	44 (52.4%)	14 (16.7%)	26 (31.0%)
>3 hours	61 (81.3%)	10 (13.3%)	4 (5.3%)
Not performed surgery	0 (0%)	0 (0%)	41 (100%)
Type of surgery			
Major	66 (84.6%)	7 (9.0%)	5 (6.4%)
Minor	46 (56.8%)	4 (4.9%)	31 (38.3%)
Not performed surgery	0 (0%)	0 (0%)	41 (100%)
Involved cardiac procedure			
YES	29 (96.7%)	0	1 (3.5%)
NO	4 (2.4%)	0	166 (97.6%)
Nephrotoxin			
Consuming nephrotoxin drugs including radiocontrast			
YES	69 (93.2%)	2 (2.7%)	3 (4.1%)
NO	2 (1.6%)	1 (0.8%)	123 (97.6%)

Regarding surgical procedures, emergency surgeries showed the highest AKI risk (96.9%), followed by elective surgeries (66.0%). Longer surgeries (>3 hours) and major surgeries were strongly associated with AKI risk (84.6% each). Cardiac surgeries also posed a substantial risk (96.7%), although a small proportion undergoing non-cardiac surgeries were still at risk.

Nurses further identified nephrotoxin exposure as a major risk factor, with 93.2% of affected patients having consumed nephrotoxic agents, including radiocontrast. However, 1.6% of patients without known nephrotoxin exposure were also flagged at risk. These findings emphasize the multifactorial nature of AKI risk and the importance of comprehensive, individualized patient assessments to improve early detection and intervention.

In this study, the Fuzzy Logic Model (FLM) was employed to interpret the risk of Acute Kidney Injury (AKI) among 200 surgical patients based on a structured risk assessment using the NURA-AKI form. The FLM approach allowed for the classification of patients into three risk categories — At Risk, Borderline, and No Risk — based on the number and severity of risk factors present.

Rather than relying on binary decision-making (yes/no or risk/no risk), the FLM incorporated multiple patient variables, such as clinical parameters, comorbidities, surgical factors, and nephrotoxin exposures. Each risk factor was assigned a membership value between 0 and 1, reflecting the degree to which it contributed to AKI risk. Using a set of predefined Fuzzy Rules, the model aggregated these membership values to determine the overall risk classification i) Patients with 4–6 risk factors (membership value closer to 1) were interpreted as “At Risk” for AKI (33.5%, n=67), ii) Patients with 3 risk factors (membership value around 0.5) were classified as “Borderline” (20.5%, n=41) and Patients with 1–2 risk factors (membership value closer to 0) were categorized as having “No Risk” (46.0%, n=92) (see **Table 6**).

Through this method, the FLM provided a graded, detailed interpretation of AKI risk, allowing for a more precise and individualized assessment of surgical patients compared to traditional binary assessments. The fuzzy classification system also better reflected the complex interplay of clinical variables influencing AKI risk, supporting early intervention strategies based on patient-specific risk profiles.

**Figure 3:**
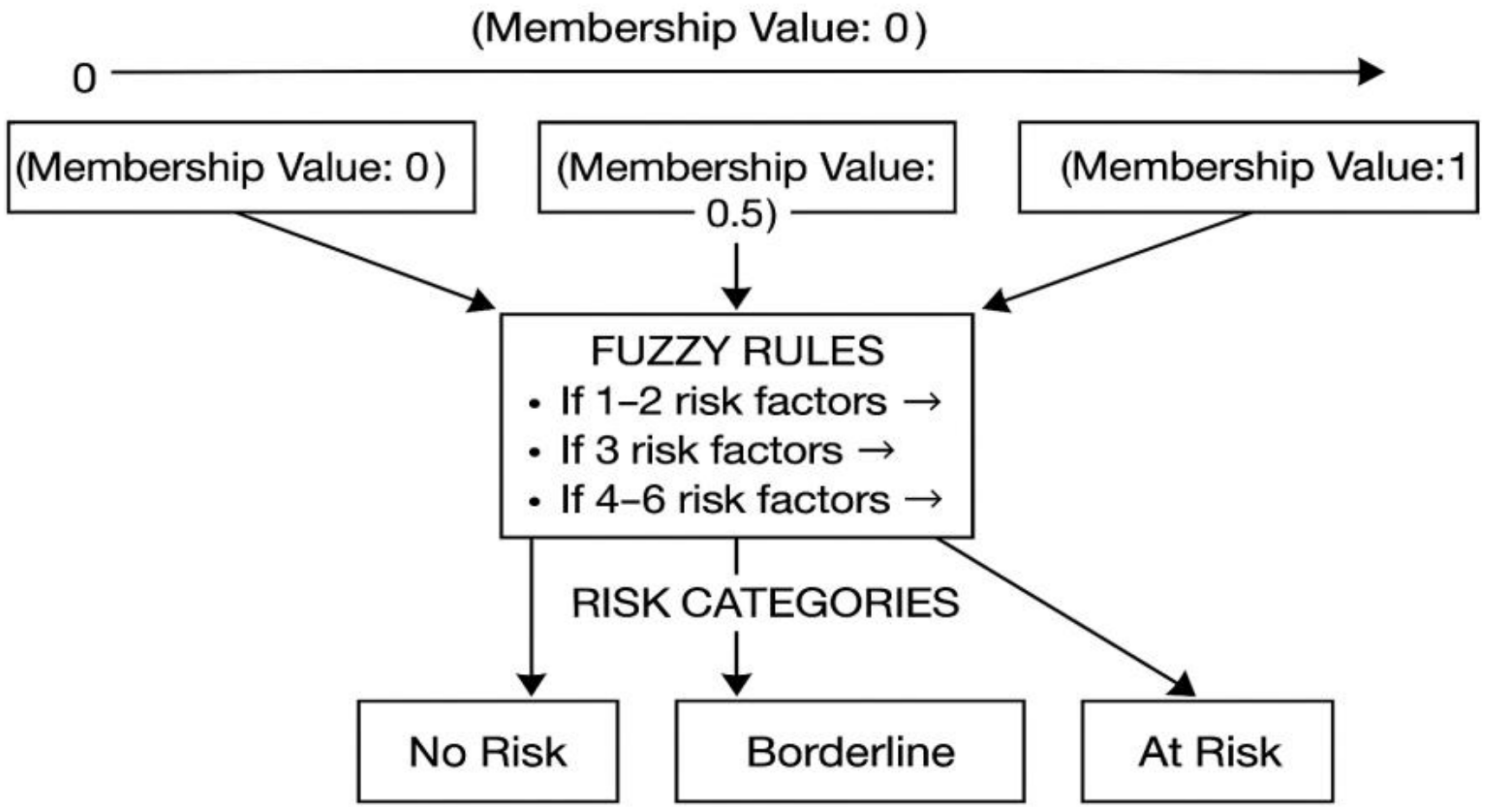
Fuzzy logic model applied to interpret AKI using nursing risk assessment tool.

**Figure 3** represents the fuzzy logic system applied in interpreting AKI risk using the Nursing Risk Assessment of AKI tool. The process begins with inputs from patient data, such as age, gender, comorbidities (e.g., hypertension, diabetes), clinical and laboratory findings (e.g., serum creatinine, urine output), surgical factors, and nephrotoxic medication use. These inputs are converted into linguistic variables (e.g., “high creatinine,” “long surgery duration”) through fuzzification. A set of predefined fuzzy rules—developed based on expert consensus—evaluates the combinations of these variables to infer degrees of AKI risk. The inference engine then applies logical operations to aggregate the risk levels, considering partial memberships across different categories. Finally, the output is defuzzified to yield a crisp classification of AKI risk: “At Risk” (33.5%), “Borderline” (20.5%), or “No Risk” (46.0%). This approach allows detailed interpretation of complex clinical data, enabling surgical nurses to make informed assessments and support timely intervention.

### 3.2 Prediction of AKI

The prediction model was statistically significant x^2^ (14, n=200) = 115.23 *p*= <0.001 suggesting that it could distinguish between those with and without the risk of AKI. Since p-value is less than 0.001 there is an association between the risk of AKI among surgical patients and the risk factors. One of the major objectives of the study was to find the association between the characteristic of the risk factors towards risk of AKI. Then the binary logistic analysis of the variables was carried out. The model explained between 45% (Cox & Snell R Square) and 61% (Nagelkerke R Square) of variance in the dependent variable and correctly classified 81.3% of cases.

As shown in **Table 6**, Logistic regression analysis was employed to model the probability of Acute Kidney Injury (AKI) occurrence as a binary outcome (AKI = 1, No AKI = 0) based on a set of clinically relevant predictor variables. These included demographic data, comorbidities, intraoperative factors, and laboratory parameters hypothesized to influence AKI risk in surgical patients. The logistic model estimates the log-odds of AKI occurrence, producing regression coefficients (β) that indicate both the direction and magnitude of association between each independent variable and the dependent outcome. The Wald test was used to assess the statistical significance of individual predictors, with p-values < 0.05 considered significant. Odds ratios (OR) with 95% confidence intervals were calculated to interpret clinical relevance. This analysis provides a robust framework for identifying modifiable and non-modifiable predictors of AKI, supporting early risk stratification and targeted interventions. 

**Table 6:** Logistic regression predicting the likelihood of risk of AKI.

Risk Factors AKI	*B*	*SE*	Wald	*df*	*p*-value	*OR*	95% CI *OR*	
							*LL*	*UL*
Patient Gender (Male)	0.742	0.397	3.496	1	0.062	2.099	0.965	4.568
Patient Age	-0.007	.013	0.253	1	0.615	0.993	0.969	1.019
Patient race (Malay)	1.661	1.505	1.218	1	0.270	5.267	0.276	100.689
Patient race (Chinese)	1.320	1.533	0.741	1	0.389	3.742	0.185	75.571
Patient race (Indian)	0.879	1.610	0.298	1	0.585	2.408	0.103	56.487
Risk Factor Comorbidity- Hypertension	0.461	.448	1.059	1	0.304	1.585	0.659	3.813
Risk Factor Comorbidity- Diabetes Mellitus	0.816	.480	2.886	1	0.089	2.261	0.882	5.794
Risk Factor Comorbidity- Chronic Liver Disease	0.510	.946	0.291	1	0.590	1.666	0.261	10.647
Risk Factor Comorbidity- Cardiovascular Disease	1.605	.590	7.414	1	0.006*	4.979	1.568	15.812
Risk Factor Comorbidity- CKD (eGRF <60ml/min per 1.73m2)	4.561	1.402	8.070	1	0.001*	9.418	3.212	35.878
Risk Factor Comorbidity- Malignancy/Tumor	1.117	.493	5.137	1	0.023*	3.055	1.163	8.022
Risk Factor Comorbidity- Sepsis/ Infection	2.115	.618	11.712	1	0.001*	8.287	2.468	27.818
Risk Factor Clinical/Laboratory SCr >26.5mmol/L within 48hours	1.869	.586	10.162	1	0.001*	6.479	2.054	20.441
Risk Factor Clinical/Laboratory Parameter Albumin level < 34g/dL or > 50g/dL	0.844	.821	1.058	1	0.304	2.326	0.466	11.618
Risk Factor Clinical/Laboratory Parameter Sodium level <135mmol/L or >145mmol/L	1.464	.907	2.604	1	0.107	4.323	0.730	25.592
Risk Factor Clinical/Laboratory Parameter Hyperkalemia >5.5mmol/L	1.348	1.155	1.361	1	0.243	3.849	0.400	37.056
Risk Factor Clinical/Laboratory Parameter Anemia Hb <10g/dL	0.463	.698	0.440	1	0.507	1.589	0.405	6.237
Risk Factor Clinical/Laboratory Parameter Urine Output <0.5ml/kg/hr for 6hours	2.289	1.295	3.125	1	0.077	9.868	0.780	124.903
Risk Factor Clinical/Laboratory Parameter Proteinuria >80g/dL	2.221	1.615	7.810	1	0.998	3.965	2.150	35.878
Emergency Surgery	-0.767	0.935	0.674	1	0.412	.464	0.074	2.900
Type of Surgery	1.103	0.526	4.397	1	0.036*	3.014	1.075	8.453
Involving Cardiac procedure	2.849	1.178	5.849	1	0.016*	17.276	1.716	173.901
Consuming nephrotoxin agents include radiocontrast	1.234	0.518	5.666	1	0.017*	3.435	1.243	9.486

*p < 0.05

The logistic regression analysis evaluated the association between various risk factors and the occurrence of Acute Kidney Injury (AKI) in surgical patients. While gender (OR=2.099, p=0.062) and diabetes mellitus (OR=2.261, p=0.089) showed increased odds of AKI, they were not statistically significant. Similarly, age, race, hypertension, and several comorbidities were not significantly associated with AKI. However, CKD (eGFR <60) showed a strong significant association (OR=9.42), indicating patients were 9.42 times more likely to develop AKI.

Among clinical and laboratory parameters, elevated serum creatinine >26.5mmol/L (OR=6.479, p=0.001), proteinuria >80g/dL, and dehydration/blood loss were statistically significant predictors. Other parameters, including hypoalbuminemia, anemia, electrolyte imbalance, and low urine output, did not reach significance. Cardiac procedures showed the strongest association (OR=17.276, p=0.016), while emergency surgery was not significant (p=0.412).

Overall, the model supports a significant association between selected risk factors and AKI occurrence, confirming the hypothesis. These findings highlight the importance of targeted risk assessment using evidence-based tools like the NURA-AKI form to aid early detection and intervention in surgical settings.

### 3.3 Sensitivity and specificity of AKI

Acute Kidney Injury (AKI) poses serious risks in surgical patients, making accurate risk prediction essential for timely intervention. Sensitivity and specificity analyses are key to evaluating predictive models, with sensitivity measuring the ability to correctly identify patients at risk (true positives) and specificity measuring correct identification of those not at risk (true negatives). In this study, sensitivity analysis revealed varied performance across risk factors. The model showed low sensitivity for age >65 years (37.7%) and moderate sensitivity for male gender (50.7%), with wide confidence intervals indicating uncertainty. Comorbidities like hypotension demonstrated very high sensitivity (98.6%), while other conditions showed moderate values. Clinical and laboratory parameters yielded consistently high sensitivities, reflecting strong model performance in detecting abnormalities linked to AKI risk. Surgical factors, particularly cardiac procedures (98.6%), and nephrotoxin exposure (85.5%) also showed high sensitivity (see **Table 7**). These findings affirm the model’s strength in minimizing false negatives and improving AKI detection. Overall, the high sensitivity across several parameters supports the model’s utility in early risk identification and enhancing clinical decision-making. 

**Table 7:** Estimates of sensitivity and specificity and their 95% CIs calculated with various risk factors.

Risk Factor	Specificity (%)	Sensitivity (%)
**Age > 65 years old**	42.7% [0.76, 1.15]	37.7% [0.78, 1.77]
**Male**	63.7% [0.98, 1.56]	50.7 [0.47, 1.00]
Comorbidity		
Hypertension	66.1% [1.18, 1.91]	60.9% [0.33, 0.73]
Hypotension	8.9% [1.19, 1.80]	98.6% [0.03, 1.46]
Diabetes Mellitus	54.0% [1.24, 1.88]	75.4% [0.27, 0.68]
Chronic Liver Disease	9.7% [1.08, 1.74]	97.1% [0.10, 1.39]
Cardiovascular Disease	33.9% [1.34, 1.89]	92.8% [0.10, 0.58]
Chronic Kidney Disease	18.5% [1.48, 1.91]	100.0%
Malignancy/ Tumor	24.2% [0.98, 1.52]	85.5% [0.24, 1.16]
Sepsis	32.3% [1.36, 1.91]	94.2% [0.08, 0.54]
Clinical and laboratory parameters		
Dehydration/ blood loss	35.5% [1.36, 1.92]	92.8% [0.09, 0.54]
SrCreatinine >26.5mmol/L within 48 hours	48.4% [1.49, 2.18]	91.3% [0.08, 0.40]
Albumin level <34 or > 50g/dL	33.1% [1.42, 1.97]	95.7% [0.05, 0.46]
Sodium level <135 or >145mmol/L	25.8% [1.39, 1.90]	97.1% [0.04, 0.54]
Potassium level >5.5 mmol/L	8.1% [1.00, 1.74]	97.1 [0.12, 1.61]
Hb level <10g/dL	30.6% [1.21, 1.75]	89.9% [0.18, 0.75]
Urine Output < 0.5ml/kg/hour for 6 hours	21.8% [1.41, 1.89]	98.6% [0.01, 0.59]
Protein level >80g/dL	8.9% [1.43, 1.80]	100.0%
Surgery procedure		
Emergency	36.3% [1.08, 1.61]	81.2% [0.32, 0.91]
Cardiac procedure	22.6% [1.42, 1.91]	98.6% [0.01, 0.58]
Nephrotoxin		
Consuming nephrotoxin drugs including radiocontrast	49.2% [1.36, 2.02]	85.5% [0.16, 0.53]

**Figure 4:**
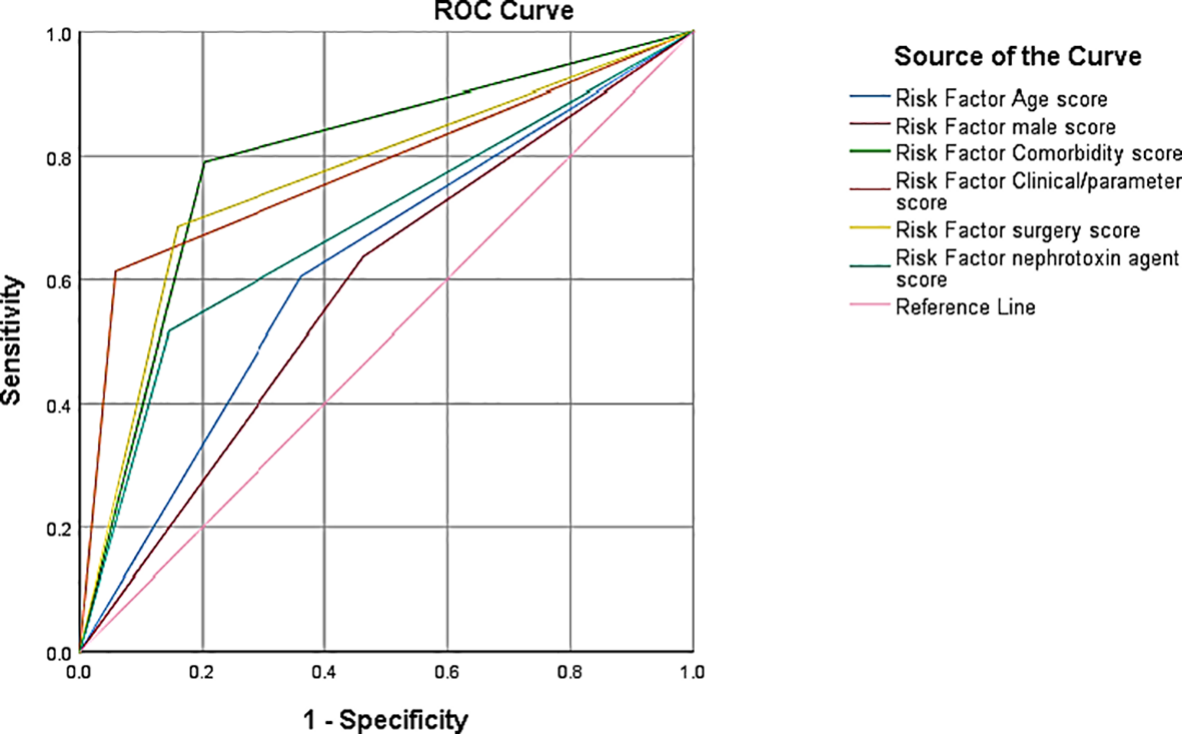
AUC-ROC curve in predicting AKI risk.

In the context of predicting the risk of AKI among surgical patients based on various risk factors, the AUC values provide depth information into the model’s discriminative ability as shown in **Figure 4**. The Area Under the Curve (AUC) values were used as a comprehensive metric to quantify the models’ ability to distinguish between patients at risk of AKI and those not at risk. The model utilizing age as a risk factor demonstrated a moderate level of separability, as reflected by an AUC of 0.621. This suggests that age contributes to the predictive accuracy but may not be as robust in differentiating AKI risk as other factors.

On the other hand, the model incorporating comorbidities exhibited a notably high AUC of 0.794, indicating a strong discriminatory power in identifying AKI risk based on the presence of comorbid conditions. Similarly, the model utilizing clinical and laboratory parameters demonstrated a robust discriminative ability with an AUC of 0.777, suggesting that these parameters significantly contribute to the accuracy of AKI risk prediction. The AUC values for gender, surgery type, and nephrotoxin agent exposure were 0.587, 0.763, and 0.686, respectively (see **Figure 4**). Thus, the null hypothesis that is no significant association between the patients AKI by predictive models and overall accuracy of positive predictions among surgical patients is rejected. These values provide valuable insights into the relative contributions of each risk factor, guiding clinicians and researchers in prioritizing factors with higher discriminative abilities for more effective AKI risk assessment in the surgical context.

## 4 Discussion

This study demonstrated the successful development and evaluation of the Nursing Risk Assessment-AKI tool for surgical nurses, which integrates a Fuzzy Logic Model (FLM) to support nurse-led identification of Acute Kidney Injury (AKI) risk among surgical patients. The form achieved a predictive accuracy of 81.3%, with sensitivity and specificity values of 83.9% and 76.3%, respectively. These findings indicate strong diagnostic performance, comparable to or exceeding existing AKI risk tools that typically require physician input or rely solely on rigid scoring algorithms (15) (37),. Notably, the use of FLM allowed detailed interpretation of risk based on multiple clinical dimensions—age, comorbidities, nephrotoxin exposure, laboratory values, and surgical parameters—offering a dynamic and adaptable alternative to binary classification models. This flexibility is crucial in surgical settings, where patient profiles are highly heterogeneous, and risk factors often overlap.

The NURA-AKI tool represents a significant departure from traditional AKI risk scoring systems such as the Kidney Disease Improving Global Outcomes (KDIGO) guidelines (38) and the Mehran risk score (39), particularly in terms of design, target users, and integration into clinical practice. While KDIGO criteria provide a well-established diagnostic framework based on serum creatinine and urine output, they require serial laboratory monitoring and are less predictive in nature. The Mehran score, primarily used for contrast-induced nephropathy, relies on multiple clinical and procedural variables often assessed retrospectively by physicians (40). In contrast, the NURA-AKI tool leverages fuzzy logic modeling to interpret complex and uncertain clinical data, enabling nurse-led dynamic risk assessment at the bedside (41). This approach enhances sensitivity and specificity, with preliminary findings showing performance comparable to or better than existing models (40).

From a nursing perspective, the usability of the Nursing Risk Assessment-AKI tool for surgical nurses was validated through structured assessments. Post-intervention data revealed that 95.7% of participating nurses agreed (68.6%) or strongly agreed (27.1%) that the tool was practical, easy to use, and fit well within their daily workflow. Confidence levels in using the form increased significantly after the education program, demonstrating that structured training combined with a user-friendly tool can enhance nurses’ clinical judgment and autonomy. These findings align with previous studies emphasizing the importance of empowering nurses in early detection roles to improve patient outcomes (14, 36, 42). Importantly, nurses were not only able to complete assessments with high accuracy but also translated AKI risk recognition into appropriate clinical action, underscoring the form’s feasibility and impact on practice.

A key innovation of this study lies in its application of the Fuzzy Logic Model to interpret AKI risk. By categorizing patients into ‘at risk’ (33.5%), ‘borderline’ (20.5%), and ‘no risk’ (46.0%) groups, the model reflected the complexity of clinical reality where risk exists on a continuum. Unlike deterministic systems, FLM accommodates the ambiguity inherent in early AKI indicators—such as fluctuating serum creatinine levels or borderline urine output—providing nurses with structured, explainable outputs to guide decision-making. This approach enhances the clarity and consistency of nursing assessments, especially in high-stakes surgical environments, and addresses the need for tools that support cognitive reasoning rather than replacing it (43). The FLM integration offers a scalable foundation for future digital nursing tools and decision support systems.

The integration of artificial intelligence (AI), particularly fuzzy logic models, into clinical decision-making processes necessitates a robust ethical framework to safeguard both patients and practitioners. While the NURA-AKI tool enhances early detection of Acute Kidney Injury (AKI), it also introduces challenges related to patient data privacy, algorithmic transparency, and potential bias. Patient information used to generate risk predictions must be managed with strict adherence to data protection regulations such as Malaysia’s Personal Data Protection Act (PDPA) to ensure confidentiality and consent (44). Moreover, algorithmic bias may arise if the model’s rule base or training data disproportionately reflects specific populations, thereby limiting the tool’s fairness and generalizability (45). To mitigate these risks, the fuzzy logic rules were developed through multidisciplinary expert consensus and validated using diverse patient profiles (46). Importantly, the tool is intended to augment but not to replace with clinical judgment, therefore nurses trained to interpret outputs contextually and escalate concerns through established protocols. Continuous evaluation, audit, and user feedback loops have been implemented to monitor for unintended consequences, including over-reliance or misclassification. As AI continues to permeate healthcare, fostering ethical AI use requires transparent model design, inclusive development processes, and strong institutional oversight to ensure that such tools reinforce, rather than compromise, patient safety and equitable care (32).

The clinical implications of this work are promising yet require cautious interpretation. The NURA-AKI tool, underpinned by a fuzzy logic model, represents an innovative approach to nurse-led, evidence-based AKI risk stratification. By enabling early identification of at-risk patients through standardized yet context-sensitive assessment, the tool has the potential to support timely interventions and reduce the likelihood of undiagnosed AKI. While these benefits may contribute to improved patient safety and more efficient perioperative care, the tool’s impact on broader clinical outcomes such as length of stay or cost savings remains to be further validated through longitudinal, multi-center studies (48). Importantly, integrating artificial intelligence methodologies like fuzzy logic into nursing practice introduces new opportunities for enhancing critical thinking and clinical autonomy; however, it also necessitates ongoing training, ethical oversight, and evaluation (49). As healthcare systems move toward precision and interdisciplinary care, tools like NURA-AKI may complement existing clinical pathways, but their successful implementation must be supported by continued refinement and rigorous validation across diverse clinical environments (47).

## 5 Limitations

This study has several limitations that warrant consideration. First, it was conducted at a single tertiary hospital (Hospital Canselor Tuanku Muhriz, Kuala Lumpur), which may limit the generalizability of findings to other healthcare settings with different patient demographics, clinical practices, or resource availability. The relatively homogeneous surgical population and specific institutional workflows could affect the external validity of the NURA-AKI tool. Second, while nurses reported high usability and confidence post-intervention, this study did not assess long-term retention or continued application of the tool in routine care. The short-term nature of the follow-up may not capture the sustainability of the tool’s impact over time. Furthermore, variations in risk factor prevalence and interpretation across different healthcare environments suggest the need for future multi-center studies to validate and adapt the fuzzy logic model for broader use. To enhance scalability and real-time utility, further development should consider integration with electronic health records (EHRs) and the design of an automated or mobile-based version of the tool. Such adaptations could facilitate immediate risk alerts and streamline nursing decision-making at the bedside, ensuring sustained improvements in AKI prevention across diverse healthcare systems.

In conclusion, the Nursing Risk Assessment-AKI tool represents a meaningful advancement in nurse-led AKI risk assessment. It enables early recognition of risk, supports informed clinical decisions, and aligns with current trends in AI-enhanced precision nursing. Embedding such tools in standard surgical workflows has the potential to reduce AKI incidence, optimize patient outcomes, and strengthen nursing autonomy in acute care. It is important to note that the NURA-AKI tool is designed to support, not replace, clinical judgment. The Fuzzy Logic Model aids in synthesizing complex risk factors to guide decision-making but does not function autonomously. Nurses are trained to interpret the tool’s output within the clinical context, and protocols emphasize that all findings are subject to professional discretion, reducing the risk of over-reliance or misinterpretation.

## Data Availability

The original contributions presented in the study are included in the article/supplementary material. Further inquiries can be directed to the corresponding author.
